# Posterior endoscopic decompression combined with anterior cervical discectomy and fusion versus posterior laminectomy and fusion for multilevel cervical spondylotic myelopathy: a retrospective case-control study

**DOI:** 10.1186/s12891-023-06713-2

**Published:** 2023-07-15

**Authors:** Lei Guo, Jiaqi Li, Fei Zhang, Yapeng Sun, Wei Zhang

**Affiliations:** Department of Spine Surgery, The Third Hospital of Hebei Medical University, Hebei, China

**Keywords:** Cervical spondylotic myelopathy, Endoscopy, Anterior cervical discectomy fusion, Laminectomy

## Abstract

**Objective:**

To compare the clinical efficacy of surgical treatment for multilevel cervical spondylotic myelopathy (MCSM) between the hybrid procedure, posterior endoscopic decompression (PED) combined with anterior cervical discectomy fusion (ACDF), and posterior cervical laminectomy and fusion (PCLF).

**Methods:**

A retrospective analysis was performed on 38 patients who received surgical treatment for MCSM from January 2018 to December 2021, including 19 cases in hybrid procedure group (13 males and 6 females), followed up for 10 to 22 (12.8 ± 10.3) months, and 19 cases in PCLF group (15 males and 4 females), followed up for 10 to 21 (11.7 ± 8.9) months. Perioperative information, including operation time, intraoperative blood loss, length of hospitalization, and complications, were compared between two groups. Visual analogue scale (VAS) of pain, neck disability index (NDI) and Japanese Orthopaedic Association (JOA) score were recorded to evaluate clinical efficacy. Cervical lordosis was calculated by radiographic examination.

**Results:**

Intraoperative blood loss, length of hospital stay were less in hybrid group than PCLF group, while operation time is longer in hybrid group, with a statistically significant difference (p < 0.05). Increased lordosis was better in hybrid group. There was no significant difference in preoperative VAS, JOA and NDI at pre-operation and final follow-up between two groups. But at post-operation and final follow-up, VAS was less in hybrid group than PCLF group (p < 0.05). There were 2 cases of neurostimulation symptoms in hybrid group, 2 cases of C5 nerve root palsy, 2 cases of subcutaneous fat necrosis and 1 case of dural tear in PCLF group, and all patients relieved with symptomatic treatment.

**Conclusion:**

The hybrid procedure of PED combined with ACDF showed satisfied clinical outcome, with less intraoperative blood loss, shorter length of hospitalization and lower post-operative neck pain than PCLF. It is an effective surgical treatment for MCSM.

## Introduction

Multilevel cervical spondylotic myelopathy (MCSM) is a common disease in elderly population, which results in worsening of neurological function and disabilities [1, 2]. Although various nonoperative managements have been proposed, surgical intervention is usually required to prevent the progression of neurological deficits [3]. In terms of pathophysiology, compression of cervical spinal cord could be from ventral and/or dorsal elements. Especially in MCSM, changes of curvature of the cervical spine and collapse of intervertebral space may lead to buckling of ligamentum flavum, which in turn exacerbates compression of spinal cord [4, 5]. Surgical methods including anterior, posterior, and combined anteroposterior approaches are widely used in treating MCSM. Anterior procedures, including anterior cervical discectomy fusion (ACDF) and anterior cervical corpectomy fusion (ACCF), showed effective and safe outcome for CSM within three segments involved. While for cases with ossification of the posterior longitudinal ligament (OPLL) or exceeding three segments involved, posterior procedures including laminectomy and laminoplasty are preferred. But there is an ongoing debate on the optimal surgical management for treatment of MCSM [6–8].

Since the first description of microendoscopic procedure in lumbar disc disease by Forley in 1997 [9], popularity of the microendoscopic technique has grown and posterior cervical microendoscopic foraminotomy was developed to fulfill decompression of cervical nerve root under direct visualization. This minimally invasive procedure was reported with advantages of reduction on intraoperative blood loss, operative time, lengths of hospitalization, postoperative pain compared with typical open surgery in cervical spondylotic disease [10, 11].

We have reported a satisfied clinical outcome of surgical method with posterior endoscopic decompression (PED), which allows for excision of dorsal compression from bony structure and ligamentum flavum [12]. This posterior endoscopic procedure showed the technical feasibility to provide an adjunctive therapy for anterior decompression surgery, which makes it possible to expand indication of ACDF for treatment of MCSM. Up to date, there is no report on comparison of clinical outcome between ACDF combined with posterior endoscopic technique and PCLF for CSM that spans more than 3 levels. The purpose of this study was to perform a short-term comparative analysis of clinical efficacy with the hybrid procedure, posterior endoscopic foraminotomy combined with ACDF, in comparison with PCLF for treatment of MCSM.

## Materials and methods

### Patient population

We reviewed all patients diagnosed with MCSM and received surgical treatment in our department between January 2018 to December 2021. The inclusion criteria included: (1) CSM involving at least three levels, (2) Either PED combined with ACDF or PCLF procedure was performed, (3) Followed up for at least 12 months. The exclusion criteria included: (1) Patients with tumor, trauma and infective diseases that were contraindicative for the above surgical treatments, (2) History of previous cervical spinal surgery, (3) Patients who refused to be enrolled in this clinical study. Hospital charts were reviewed for demographic data, operation time, intraoperative blood loss, length of hospitalization and surgical complications. This clinical study was conducted with approval by the Ethics Committee of Third Hospital of Hebei Medical University. Written informed consent was obtained from all patients. All the procedures were carried out in accordance with The Code of Ethics of the World Medical Association (Declaration of Helsinki) for human experiments.

### Surgical technique

All surgeries were performed by a single senior surgeon in our department. The operative levels were determined according to patients’ symptoms, signs, radiological images, and electromyography in necessity. The surgical approaches were determined by the surgeon’s decision. For patients with three-level cervical spondylotic myelopathy, with both anterior compression from discs and posterior compression from ligamentum flavum, the hybrid procedures were performed. Firstly, two-level ACDF were selected for decompression of anterior elements, including herniated disc or ossification of annulus, and then PED were selected for decompression of posterior elements, including hypertrophy or calcification of the ligamentum flavum. For multilevel exceeding three segments CSM or cervical severe stenosis, posterior cervical laminectomy was used to fulfill a sufficient decompression of spinal cord and nerves.

### Hybrid surgery

**Fig. 1:**
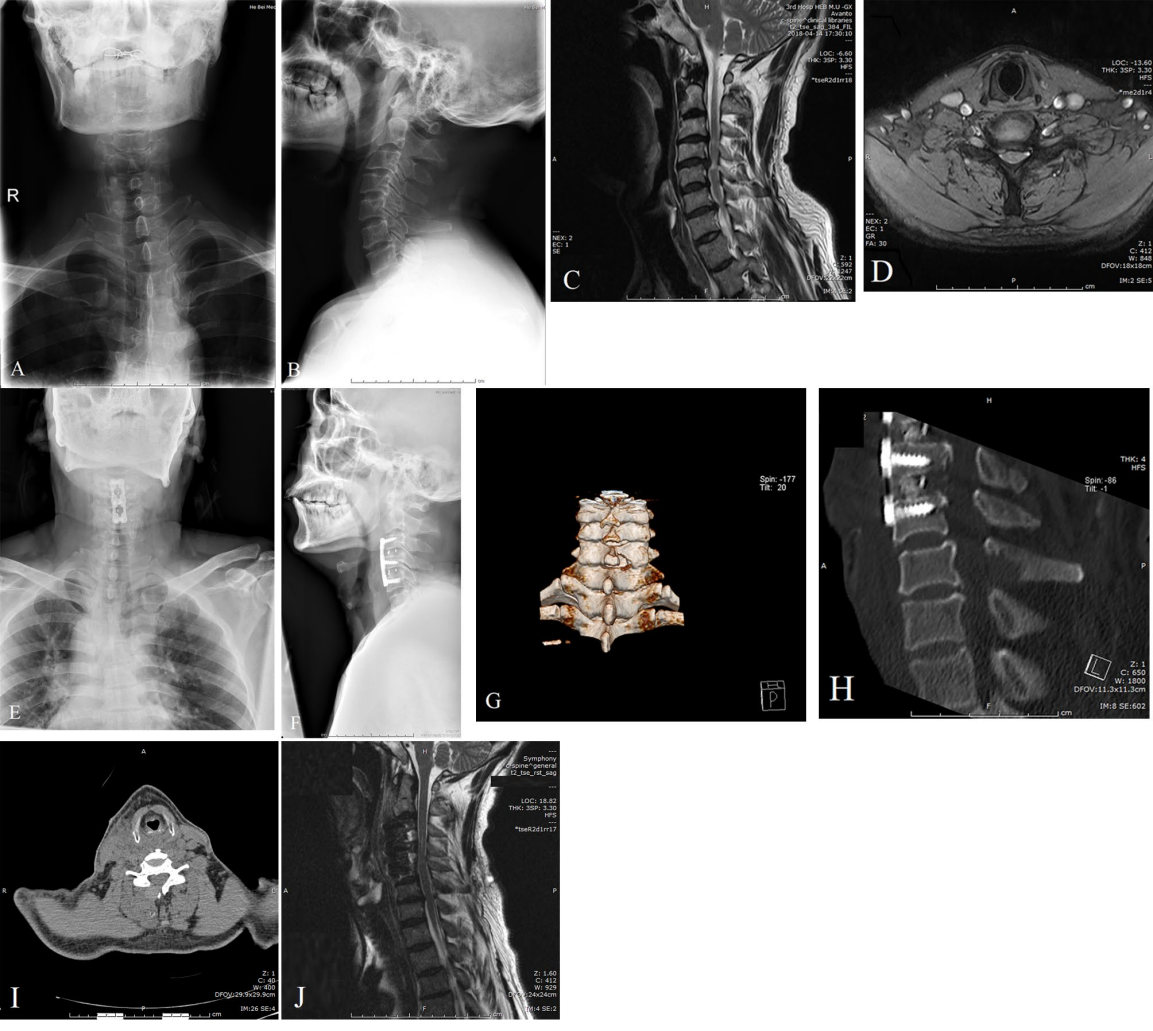
A 52 years old male patient diagnosed with MCSM underwent the hybrid surgery. (**A** and **B**) Preoperative anterior and lateral X-ray radiograph of cervical vertebrae. **(C)** Preoperative MRI indicate the compression from disc herniation (C3-5 and C6-7). **(D)** Cross-section of MRI at C6-7 level. (**(E)** and **(F)**) Anterior and lateral X-ray radiograph of cervical vertebrae after C3-5 ACDF surgery. (**G** and **H**) Three-dimensional CT scan of cervical vertebrae after adjunctive PED surgery. **(I)** Cross-section of CT scan at C6-7 level. **(J)** Postoperative MRI indicate a sufficient decompression of cervical spinal cord (C3-5 and C6-7)

**Fig. 2:**
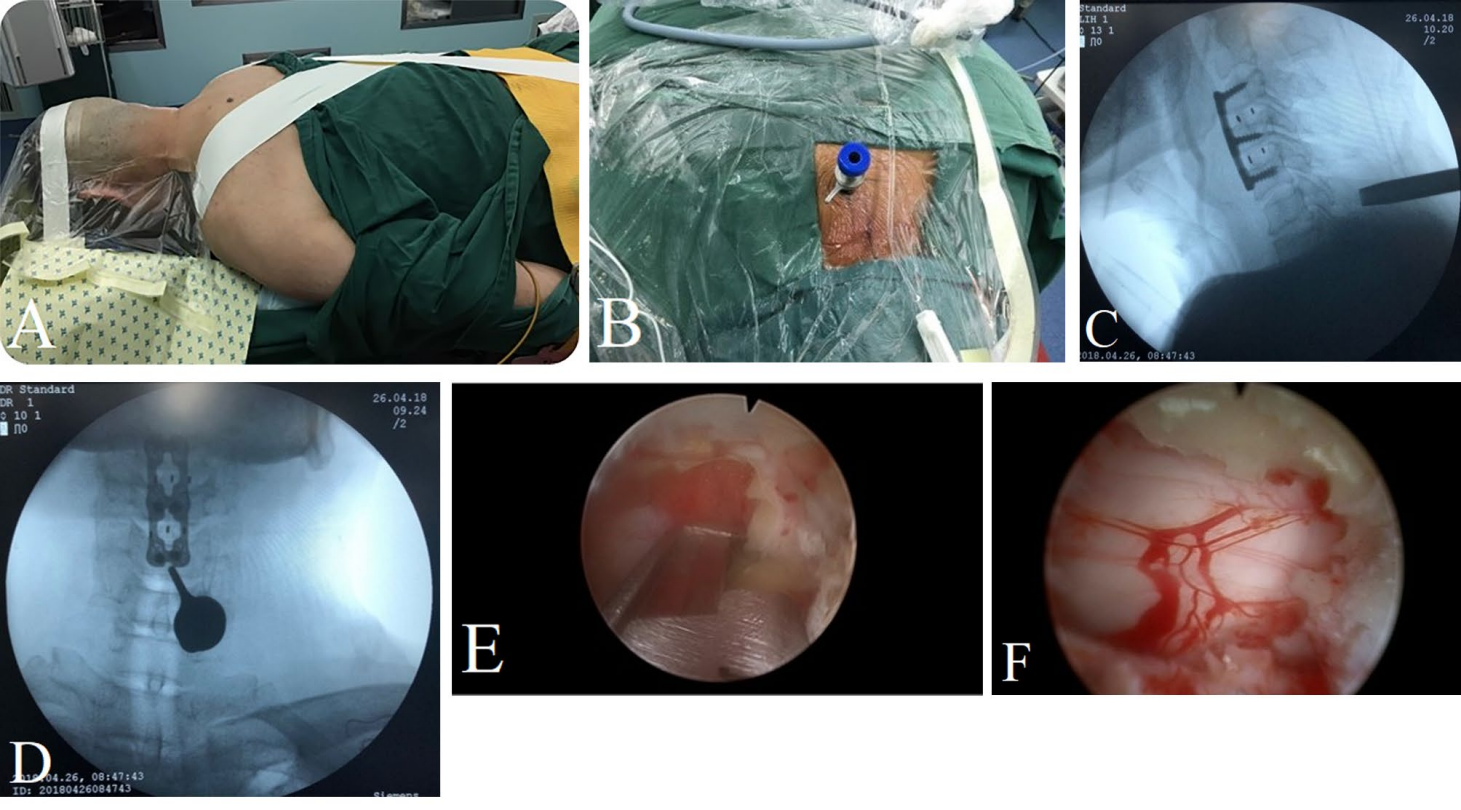
Posterior endoscopic decompression procedure. **(A)** Patient was placed in the prone position under general anesthesia. **(B)** The working channel was placed at the corresponding level with an 8–10 mm incision. (**C** and **D**) Intraoperative lateral and anterior X-ray radiograph of cervical vertebrae confirmed position of the working channel. **(E)** Decompression of dorsal elements by a rongeur. **(F)** Directly visual decompression of spinal cord

All patients in hybrid group underwent a standard two-level anterior cervical discectomy and fusion surgery under general anesthesia. First, patients were placed in supine position. Incision was performed using a right-side approach at the target level. After discectomy, cages (Weigao Inc. Shandong, China) filled with autologous bone were inserted into the resected intervertebral space, and an anterior plate (Weigao Inc. Shandong, China) was utilized (Fig. 1). The incision was sutured layer by layer. A drainage tube was applied and removed at 48 h post operation. After finishing the ACDF procedure, each patient was placed in a prone position. An adhesive tape was used for fixation of head to the operating table. A working channel and endoscope were placed targeting the “V” point of the anatomical landmark, then a rongeur and drill were used to fulfill decompression of dorsal elements, including partial lamina, ligamentum flavum, and articular process should be carefully preserved with more than 50% to the outside. As we previously reported [12], directly visual decompression of spinal cord and nerve root could be well performed through unilateral approach (Fig. 2). While for some cases with severe cervical stenosis, bilateral approach was used.

### PCLF surgery

**Fig. 3:**
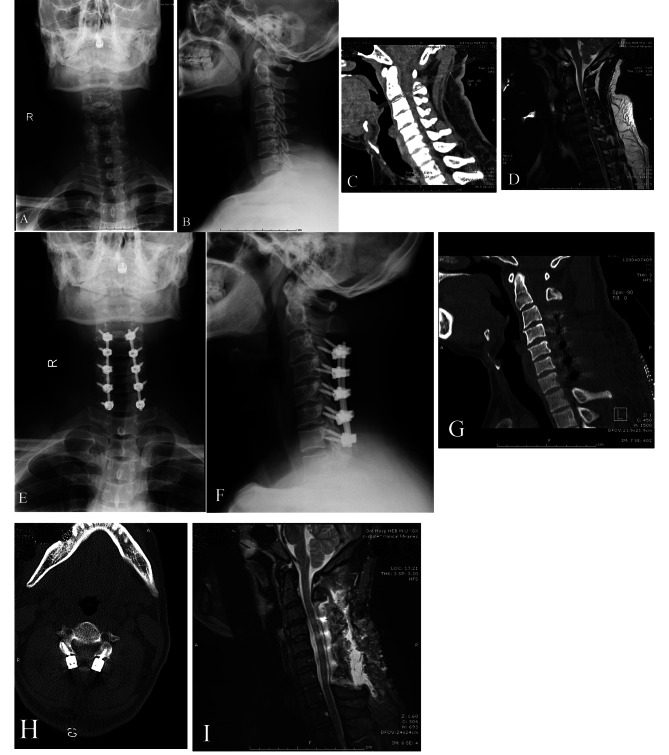
A 38 years old male patient diagnosed with MCSM underwent PCLF surgery. (**A** and **B**) Preoperative anterior and lateral X-ray radiograph of cervical vertebrae. (**C** and **D**) Preoperative CT and MRI indicate the compression from osteophyte and cervical stenosis. (**E** and **F**) Anterior and lateral X-ray radiograph of cervical vertebrae after C3-7 PCLF surgery. (**G** and **H**) Postoperative three-dimensional CT scan of cervical vertebrae indicate complicate laminectomy and accurate lateral mass screw fixation. **(I)** Postoperative MRI indicate a sufficient decompression of cervical spinal cord at C3-7 levels

Under general anesthesia, patients in PCLF group underwent a standard C3-C7 posterior laminectomy and lateral mass screw fixation (Weigao Inc. Shandong, China) in prone position. A skull traction device, Mayfield headstock, was used for fixation of head during the procedure. A midline incision was selected and bilateral paravertebral muscle strip dissection were performed to expose the posterior structure of cervical vertebrae. A total of 10 lateral mass screws were inserted to the C3-C7 lateral mass, and 2 rods were connected bilaterally. The spinous process and lamina from C3 to C7 were resected carefully to fulfill posterior decompression of spinal cord. Autologous bone from the resected C3-C7 spinous process and lamina were used for posterolateral bone graft fusion. The incision was sutured layer by layer, and a drainage tube was kept until the volume of drainage was less than 50ml in 24 h (Fig. 3).

### Clinical evaluation

Perioperative information, including operation time, intraoperative blood loss, length of hospitalization, and complications were recorded. Visual analogue scale (VAS) was used to evaluate pre- and postoperative neck pain. Neck disability index (NDI) and Japanese Orthopaedic Association (JOA) score of cervical spine were recorded to evaluate clinical efficacy.

### Radiological evaluation

Cervical lordosis was assessed by radiological parameters calculated on lateral X-ray images preoperatively, instant postoperatively and at final follow-up. Cobb angle were calculated as the angle formed between the superior endplate of C2 vertebrae and the inferior endplate of C7 vertebrae. Computed tomography (CT) and magnetic resonance imaging (MRI) of cervical spine were performed for diagnosis and postoperative evaluation.

### Statistical analysis

All numeric data were presented as mean ± standard deviation. Statistical analysis was conducted using SPSS v22.0 software (IBM Inc., Chicago, Illinois). Parameters were compared using Chi-square and Student t tests. A p-value < 0.05 was considered statistically significant.

## Results

There were 19 patients in hybrid group (13 males and 6 females) and 19 patients in PCLF group (15 males and 4 females). The mean age of patients in hybrid and PCLF group was 58.4 ± 11.1 years and 57.3 ± 9.2, respectively. Patients were followed up for 10 to 22 (12.8 ± 10.3) months in hybrid group, and 10 to 21 (11.7 ± 8.9) months in PCLF group. There was no statistical difference in age, gender, follow-up duration between the two groups. The average anterior fused segment was 2 levels in hybrid group, with an additional PED surgery. All patients in PCLF group received posterior C3-C7 laminectomy, lateral mass screw fixation and fusion.

**Fig. 4:**
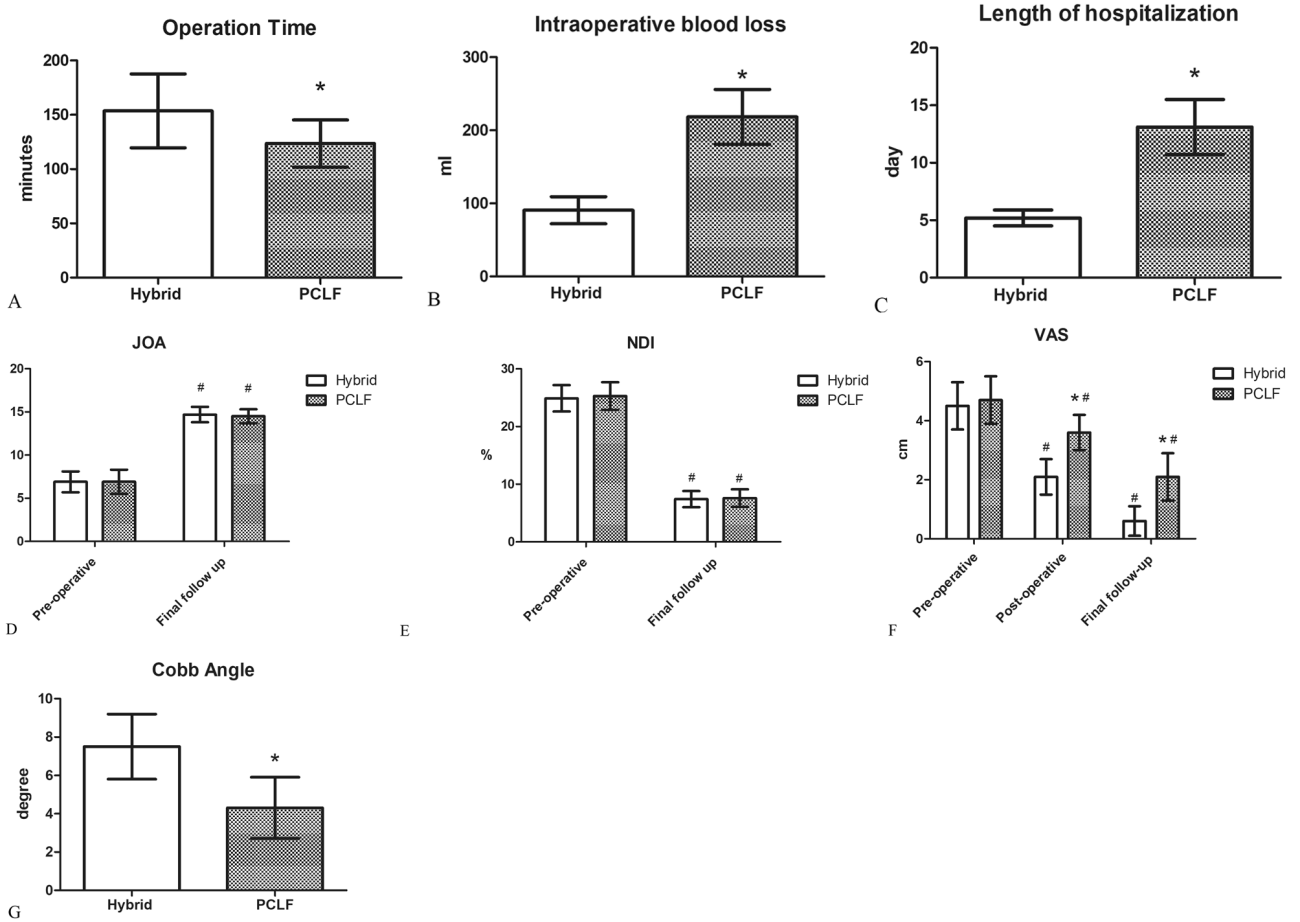
Comparison of clinical outcomes between two groups. **(A)** Comparison of operation time between two groups, * with a statistically significant difference between two groups, p < 0.05. **(B)** Comparison of intraoperative blood loss between two groups, * with a statistically significant difference between two groups, p < 0.05. **(C)** Comparison of length of hospital stay between two groups, * with a statistically significant difference between two groups, p < 0.05. **(D)** Comparison of JOA score before operation and at final follow up, # with a statistically significant difference compared with preoperative values in each group, p < 0.05. **(E)** Comparison of NDI before operation and at final follow up, # with a statistically significant difference compared with preoperative values in each group, p < 0.05. **(F)** Comparison of VAS score before operation, post operation and at final follow up, * with a statistically significant difference between two groups, p < 0.05. # with a statistically significant difference compared with preoperative values in each group, p < 0.05. **(G)** Comparison of cobb angle between two groups, * with a statistically significant difference between two groups, p < 0.05

In hybrid group and PCLF group, operation time, intraoperative blood loss and length of hospitalization were (153.6 ± 34) min, (123.6 ± 21.7) min, (90.6 ± 18.4) ml, (218.3 ± 37.5) ml, (5.2 ± 0.7) days, (13.1 ± 2.4) days, respectively. Cobb angle at final follow-up were (7.5 ± 1.7) °in hybrid group, and (4.3 ± 1.6) ° in PCLF group. Preoperative and three days postoperative VAS score were (4.5 ± 0.8), (2.1 ± 0.6) in hybrid group, (4.7 ± 0.8), (3.6 ± 0.6) in PCLF group, and decreased to (0.6 ± 0.5), (2.1 ± 0.6) in hybrid group and PCLF group at final follow up, respectively. Preoperative NDI and JOA score were (24.9 ± 2.3), (6.9 ± 1.2) in hybrid group, (25.3 ± 2.4), (6.9 ± 1.4) in PCLF group, and improved to (7.4 ± 1.4), (14.7 ± 0.9) in hybrid group, (7.6 ± 1.5), (14.5 ± 0.8) in PCLF group at final follow-up. There was no statistical difference in preoperative scores of JOA, NDI and VAS between the two groups. Improvement in JOA scores and NDI were observed at final follow up, with a statistically significant difference in each group, compared with the preoperative values (P < 0.05), but there was no statistical difference at final follow up between the two groups (P > 0.05). Figure 4 shows there was a statistical difference between the two groups in terms of operation time, intraoperative blood loss, length of hospitalization, VAS scores post operation and at final follow up, cobb angle at final follow up (P < 0.05).

In hybrid group, 2 cases developed postoperative neurostimulation symptoms, and relieved with conservative treatment. There was no hemotoma or hoarseness occurred in hybrid group. In PCLF group, 2 cases developed C5 nerve root palsy and recovered with neurotrophic drugs at 6 months post-operation. 2 cases developed delayed wound healing and resolved with surgical debride. 1 case occurred with dural tear during decompression procedure, and treated with a tight suture and positive pressure drainage after surgery. There was no death, pseudoarthrosis, reoperation and implant complications occurred during perioperative period and follow up in both two groups.

## Discussion

Since anterior approach surgery alone does not afford the ability of adequate decompression of posterior canal space, it is still controversy on surgical strategy for treatment of MCSM, especially when both ventral and dorsal compression occurred. Cervical laminectomy with fusion is a commonly utilized surgical option for treatment of MCSM and ossification of posterior longitudinal ligament (OPLL). Common practice for surgical planning of CSM is anterior operation for 1 or 2 levels, and posterior for 3 or more. Several studies compared clinical outcome between anterior and posterior decompression procedure for MCSM and showed similar efficacy with regard to functional improvement, disability and quality of life [13, 14]. For rates of complications, Veeravagu A et al. found greater rates of postoperative complications with posterior decompression procedure, including cardiac complications, pulmonary complications, wound complications and deep vein thrombosis (DVT), especially in the elderly population [15]. Badhiwala JH et al. found posterior decompression and fusion was associated with longer length of stay, greater rates of general medical complications, and higher hospital charges and costs than ACDF [16].

Posterior cervical endoscopic technique was first introduced by Rutten et al. [17] and since then the minimally invasive surgery of cervical endoscopy has been reported to be with a similar clinical outcome compared with traditional open surgeries for treatment of cervical spondylotic diseases [18, 19]. We have reported our experience of treatment for CSM and cervical spondylotic radiculopathy (CSR), using PED surgery combined with ACDF, and obtained satisfactory clinical outcome. While up to now, no study has compared clinical outcome between the posterior endoscopic decompression surgery combined with ACDF, and traditional laminectomy with fusion, for treatment of MCSM. Thus, the purpose of this study was to ascertain the impact of the hybrid procedure on perioperative safety, clinical efficacy and rates of surgical complications, and provide evidence of effectiveness over the posterior decompression and fusion procedure for treatment of MCSM.

There was significant improvement in clinical outcome scores (JOA, NDI) in each group at final follow up, with no significant difference between the two groups. The results showed a comparatively similar clinical efficacy of the hybrid procedure, compared to PCLF surgery. The adjunctive PED procedure combined with ACDF was approved to be an alternative on surgical plan for MCSM exceeding 3 levels.

The advantage of hybrid surgery is decreased VAS scores at three days post-operation and final follow up, in comparison with PCLF surgery. Especially at early stage of recovery, there was a significant difference on VAS at three days post-operation between two groups. This might be resulted from the extensive dissection in posterior laminectomy surgery, which might pose an impact on post-operative pain. While in hybrid group, both anterior and posterior less invasive procedure contributed to lower VAS scores. In our study, continuous improvement on VAS were found in both groups, and there was a significant difference between two groups at final follow up. Kristof et al. reported similar axial neck pain in both anterior and posterior groups [20]. The controversy may be resulted from anterior decompression surgeries that were selected. In their study, the anterior group consisted of multilevel ACCF, more commonly two- or three-level ACCF were performed. While in our study, with the assist of posterior endoscopic surgery, number of anterior fusion segments can be reduced. All patients were performed with a two-level ACDF and an additional PED surgery in a minimally invasive method, thus resulted in reduction of VAS scores at post-operation and final follow up.

In PCLF group, significantly higher blood loss was found compared with hybrid group. Technically, ACDF and PED are relatively minimally invasive procedures. With skilled training and experience, intraoperative blood loss in hybrid procedure can be well controlled. While posterior laminectomy with fusion requires extensive dissection of paravertebral muscle and bony structure, which resulted in a greater amount of intraoperative blood loss. This was consistent with the finding of Seng et al. [21]. Operation time was longer in Hybrid group. This may because of the surgical position needed to be changed during the hybrid procedure. The patients underwent the ACDF surgery in supine position firstly, and then changed to prone position to fulfill posterior endoscopic decompression. This part of procedure took up a portion of operation time. Notwithstanding, two-level ACDF and single level PED procedure are both technically faster operation. While patience is required in the extensive surgical exposure, accurate and careful placement of lateral mass screws, and bilateral laminectomy in PCLF procedure.

Posterior decompression surgery is one of the most commonly used surgical interventions for multilevel CSM, especially for cases with more than three segments involved. Posterior surgeries, including laminoplasty and laminectomy with fusion, require extensive dissection of paraspinal muscles, which may result in postoperative axial neck pain, and decrease in cervical lordosis and range of motion (ROM). Pan FM et al. reported an average incidence of C5 nerve root palsy after posterior cervical decompression surgery was 7.8% [22]. In our study, 2 cases (10.5%) developed C5 nerve root palsy in PCLF group, and the symptoms were full-relieved with conservative treatment within 6 months post operation. The most likely cause of this complication was posterior drift of the spinal cord with tethering of nerve root. Other approach-related complications included incision infection, delayed wound healing, and decompression radiculopathies. Kristof RA et al. reported the rate of wound infection was 6.5% in posterior decompression surgery, and rate of radiculopathy after decompression was 19.6%. All radiculopathies were slightly and reversible within months to several years [20]. In our study, there was no wound infection and decompression radiculopathy occurred in PCLF group. This difference could be attributed to the small sample size of our study. 1 case developed intraoperative dural tear and fixed with tightly suture of dural sac. 2 cases developed delayed wound healing, and treated with regular change of surgical dressing.

Traditional ACDF was recognized as the gold standard for treatment of CSR and for cases with 1- or 2-level CSM, but it’s efficacy for multilevel CSM is controversial [23–25]. Hardware failure is one of the most frequent complications following anterior cervical surgeries, and the number of fusion segments were positively associated with the incidence of hardware failure, from 1 to 4% in one level, and up to 16–71% in three and more levels [20]. Meanwhile, several studies found ACDF was associated with more approach-related complications, including hoarseness, dysphagia and post-operative hematoma [21, 26]. Other complications, including adjacent segmental degeneration (ASD), graft subsidence, loss of intervertebral height and ROM, have been reported in ACDF with long-term follow up [27–29]. In our study, there was no hardware failure or pseudoarthrosis in hybrid group at final follow-up. This may benefit from the selection of a two-level ACDF for anterior decompression in hybrid group, and long-term of follow up is needed to observe clinical outcome and safety of this hybrid technique.

Complications in PED surgery are frequently represented with aggravated pain and numbness, decreased muscle strength, and disc herniation recurrence. Haijun M, et al. reported a lower complication rate in PED surgery using Delta system than that of traditional key-hole surgery. The symptoms of aggravated pain and numbness, and decreased muscle strength were fully relieved with analgesic medicine and nutritional nerve drugs within 2 weeks after surgery [30]. In our study, the PED surgery was performed by Delta system and a larger surgical field of view was obtained. Thus, it was easier to distinguish and protect nerve root from compression component. Meanwhile, the PED surgeries in our study were performed under general anesthesia, which reduced pain-related high blood pressure and intraoperative blood loss could be well-controlled, and contributed to reduce stimulation of nerve root through the working channel.

### Limitation

This is a retrospective case-control study with a short-term follow up, and sample size in this clinical study was relatively small. Further high-quality randomized clinical studies with long-term follow up are needed to evaluate the clinical efficacy and safety of the hybrid procedure, and help provide evidence-based proof for optimal surgical strategy of MCSM treatment.

## Conclusions

Compared with PCL surgery, the hybrid procedure of posterior endoscopic foraminotomy combined with ACDF showed good clinical efficacy, with less intraoperative blood loss, shorter length of hospitalization and lower risk of surgical complications. It is a safe and effective surgical method for treatment of multilevel CSM.

## Data Availability

The datasets used and analyzed during the current study are available from the corresponding author on reasonable request.

## Abbreviations

PED: Posterior endoscopic decompression
ACDF: Anterior cervical discectomy and fusion
PCLF: Posterior cervical laminectomy and fusion
MCSM: Multilevel cervical spondylotic myelopathy
CSR: Cervical spondylotic radiculopathy
ACCF: Anterior cervical corpectomy fusion
OPLL: Ossification of the posterior longitudinal ligament
JOA: Japanese Orthopaedic Association
NDI: Neck disability index
VAS: Visual analogue scale
CT: Computed tomography
MRI: Magnetic resonance imaging
DVT: Deep vein thrombosis
ROM: Range of motion
